# Association of dietary inflammatory index and the SARS-CoV-2 infection incidence, severity and mortality of COVID-19: a systematic review and dose-response meta-analysis

**DOI:** 10.1186/s12937-024-00927-3

**Published:** 2024-02-20

**Authors:** Xuanyu Hao, Shiwen Li, Yanmin Yang, Huixu Dai, Yumeng Yan, Dongyang Li

**Affiliations:** 1 Department of Gastroenterology, Shengjing Hospital of China Medical University, Shenyang, Liaoning, 110004 China; 2 Department of Healthcare-associated Infection Management, Department of Pulmonary and Critical Care Medicine, Shengjing Hospital of China Medical University, Shenyang, Liaoning, 110004 China; 3 Department of Gerontology and Geriatrics, Shengjing Hospital of China Medical University, Shenyang, Liaoning, 110004 China; 4 Department of Clinical Epidemiology, Shengjing Hospital of China Medical University, Shenyang, Liaoning, 110004 China; 5 Department of Clinical Nutrition, Shengjing Hospital of China Medical University, Shenyang, Liaoning, 110004 China; 6 Department of Urology, Shengjing Hospital of China Medical University, Shenyang, Liaoning, 110004 China

**Keywords:** COVID-19, Dietary inflammatory index, Incidence, Severity, Mortality

## Abstract

**Background:**

Several studies have reported the association between dietary inflammatory index (DII) and the SARS-CoV-2 infection risk, severity or mortality of COVID-19, however, the outcomes remain controversial.

**Objective:**

We sought to examine whether a dose-response association of DII and SARS-CoV-2 infection exists.

**Design:**

A dose-response meta-analysis was performed to investigate the association of DII and SARS-CoV-2 infection. We conducted a systematic search of PubMed, Embase and Web of Science up to March 15th, 2023. The odds ratios (OR) of DII and COVID-19 risk and severity were computed.

**Results:**

Totally, 5 studies were included (1 from UK and 4 from Iran), consisting of 197,929 participants with 12,081 COVID-19 cases. Although there was heterogeneity among studies, the results indicated that higher DII was independently related to higher SARS-CoV-2 infection incidence (OR = 1.57, 95% CI: 1.14, 2.17) and COVID-19 severity (OR = 1.11, 95% CI: 1.07, 1.15) but not COVID-19 mortality (risk ratio = 1.13, 95% CI: 1.00, 1.27). The incidence of SARS-CoV-2 infection increased by 31% for each 1-point increase in the E-DII (OR = 1.31, 95% CI: 1.20, 1.43).

**Conclusions:**

This meta-analysis suggests that an elevated DII score is associated with increased SARS-CoV-2 infectious risk and severity of COVID-19. There were not enough studies on COVID-19 mortality. Further large prospective studies in different countries are warranted to validate our results.

**Supplementary Information:**

The online version contains supplementary material available at 10.1186/s12937-024-00927-3.

## Introduction

Since the initial cases of COVID-19 were reported in 2019, the world has been grappling with a swift global pandemic [1]. While vaccines can shield against severe disease and ideally contribute to herd immunity, the emergence of new SARS-CoV-2 variants (such as Omicron, XBB lineage, etc.) is likely to result in fresh outbreaks due to heightened infectivity, virulence, or enhanced potential for immunological evasion [2, 3]. COVID-19 manifests through distinct inflammatory pathways, immune responses, and potentially devastating cytokine storms [4, 5]. Being a crucial aspect of life, a high-quality daily diet and patterns have been reported to correlate with a reduced risk of SARS-CoV-2 infection and hospitalization [6]. In contrast, malnutrition serves as an ominous prognostic sign in patients hospitalized with COVID-19 [7]. Studies have shown an inverse relationship between the Mediterranean diet and the risk of COVID-19, suggesting its potential utility in mitigating COVID-19-induced inflammation [8, 9]. Food antioxidant supplements, such as vitamin C and vitamin D3, exhibit a strong correlation with decreased severity and mortality in COVID-19, as indicated by randomized controlled trials [10, 11]. Diet may participate in the inflammatory response in patients with COVID-19 [12]. So what is the specific effect of anti-inflammatory diet or pro-inflammatory diet on COVID-19?

The Dietary inflammatory index (DII) is a quantitative tool for assessing diet inflammatory potential based on the inflammatory factors from daily intake from a multiple items food frequency questionnaire (FFQ) [13]. The Energy-adjusted DII (E-DII) primarily concentrates on energy intake, and elevated DII/E-DII levels are supported by inflammatory markers like C-reactive protein [14]. Numerous studies have highlighted the crucial role of DII in assessing the inflammatory potential of diets and predicting the risk of chronic diseases, such as cardiovascular diseases or depression [15, 16]. We have also previously reported a linear dose-response relationship between DII and the risk of human cancer [17]. Therefore, to prevent and reduce the severity or mortality of COVID-19, DII may serve as a reminder for individuals to maintain healthy eating habits.

Regarding the relationship between an inflammatory diet and COVID-19, researchers have discovered that a high-quality diet can lower the levels of inflammatory factors [18]. Research has demonstrated the relevance of the Mediterranean diet to a reduced risk of SARS-CoV-2 infection and improved outcomes in patients with COVID-19 [19]. Additionally, diets rich in antioxidants, such as vitamin D, with immunomodulatory potential, may contribute to prophylactically mitigating the severity of COVID-19 [20]. Meanwhile, an unhealthy diet should not be neglected as a potential source of inflammation, given that chronic inflammation has adverse effects on health. The Western diet, characterized by high consumption of processed foods, may induce hyperglycemia and hyperlipidemia. Diets with different DIIs may exhibit different effects on regulating the level of inflammation in the body, which in turn affects the rate and severity of COVID-19 infections. Li et al. found that hyperglycemia is associated with a high risk of mortality in patients with severe COVID-19 [21, 22]. Furthermore, the Western diet may be associated with adaptive immunity impairment in patients with COVID-19, potentially contributing to aggravation of the disease [23]. Over the past decades, certain strategies, such as the Healthy Eating Index and Alternative Healthy Eating Index, have been investigated for their association with the risk of all-cause mortality [24, 25]. The DII, a comprehensive, reproducible and quantitative method of FFQ calculation, can recognize the exact inflammatory potential of diet. The low DII items contain deleted n-3 fatty acids, β-carotenes, phytochemicals, and plant fiber, whereas the Western diet (high DII) means a high consumption of red and processed meat, trans-fatty acid or saturated fat acid [26].

To date, the results from studies on DII and COVID-19 remain inconclusive and controversial. For instance, individuals with higher DII had a higher SARS-CoV-2 infectious risk and mortality of COVID-19 in a UK-biobank cohort [27]. However, the results from Tavassoli and Cols indicated no association between DII score and SARS-CoV-2 infection incidence (OR = 1.08, 95% CI: 0.92–1.27) [28]. Here we systematically summarize the currently available evidence on DII and the incidence of SARS-CoV-2 infection, severity and mortality of COVID-19. Meta-analyses were performed on the incidence and severity, and a dose-response analysis was conducted on the incidence. Additionally, meta-regression and sensitivity analysis were carried out to assess stability. To date, this is the first meta-analysis concerning dietary-related inflammation and COVID-19, as far as we know.

## Methods

The study was preregistered with PROSPERO under project number CRD42023407410.

### Search strategy

According to the guidelines of the Preferred Reporting Items for Systematic Reviews and Meta-Analyses (PRISMA), we carried out this study [29]. We comprehensively searched for relevant literature published in PubMed, Embase and Web of Science from January 1st, 2020 to March 15th, 2023. The main terms were as follows: (‘pro-inflammatory diet’ OR ‘dietary inflammatory index’ OR ‘anti-inflammatory diet’ OR ‘inflammatory potential intake’) AND (‘COVID-19’ OR ’SARS-CoV-2 ’) AND (‘risk’ OR ‘incidence’ OR ‘odds’ OR ‘hazards’ OR ‘mortality’ ). There were no language restrictions, and we manually searched reference lists for additional relevant publications.

### Inclusion and exclusion criteria

The eligible criteria were under the guide by the Population, Intervention, Comparison, Outcome (PICO) question: In general adult population (P), DII or EDII score (I), higher DII/E-DII group compared to lower DII/E-DII group (C), and COVID-19 incidence (primary outcome), severity or mortality (secondary outcomes) (O). The exclusion criteria were: (1) Reporting DII during or after the pandemic; (2) Repeated publications; 3.Less than 30 participants; 4. Laboratory articles, non-human animal studies, or review articles.

### Assessment of study quality

Two authors (X.H. and S.L.) independently screened titles and abstracts, meanwhile, they evaluated the methodological quality of potential studies using the Newcastle–Ottawa Quality Assessment Scale (NOS) tool [30]. When the NOS score was none less than 7, the study was regarded as high quality. If the two authors had a disagreement, a third author made the final decision (D.L.).

### Data extraction

After a full-text review, we extracted the following variables from each included study: the first author’s name, publication year, study design, country of the participants, sample size (number of COVID-19 patients), age, sex, multivariate adjustments, outcomes and ORs with 95% CI. Only the multivariate ORs were adopted for meta-analysis because of the higher objectivity than univariate ORs. The corresponding author was contacted for further information under the data-deficiency circumstance.

### Statistical analysis

We synthesized odds ratios (ORs) with 95% confidence intervals (CIs) using the meta-analysis method to investigate the association between the Dietary Inflammatory Index (DII) and the incidence and severity of COVID-19. The DII scores from eligible studies were continuous variables or categorical (the highest quartile versus the lowest). Heterogeneity among studies was assessed using the Cochrane Q test (*P* value < 0.10 represented heterogeneity) and Inconsistency(*I*^*2*^ value > 50% indicated heterogeneity) [31]. Due to potential heterogeneity, the DerSimonian and Laird inverse variance random-effects model was applied to calculate pooled results [32]. Subgroup analyses were conducted to explore the causes of heterogeneity, considering study design, DII or E-DII, study quality and ethnic differences. In addition, we used sample size and sex proportion as co-variates to do a meta-regression.

Subsequently, we conducted a dose-response analysis to examine the relationship between E-DII and the incidence of COVID-19. The OR, 95% CI and corresponding dose values for each category of E-DII (lowest to the highest) were extracted. We assigned the midpoint of each category when the dose values were unavailable [33]. If the highest (lowest) category was open-ended, we adopted the lower end value of the category and added (reduced) the closest neighboring category dose interval value [34]. We applied the ‘glst’ command and a random-effect four knots cubic spline (non-linear) model in STATA software to do a conservative dose-response analysis [35, 36]. Four dose knots at 5% (0), 35% (dose1), 65% (dose2) and 95%(dose3), in the dose range were generated before evaluating the dose-response via the restricted cubic spline method [17].

For evaluating the reliability of the main pooled outcome, a sensitivity analysis was conducted by removing a single study in turn. Egger’s and Begg’s tests, along with funnel plots, were adopted to assess publication bias [37]. A *P* value > 0.1 indicated no potential publication bias. All statistical analyses mentioned above were performed using STATA 12.0 software (Stata Corporation, TX, USA). A two-sided *P* value < 0.05 was considered statistically significant.

## Results

### Study search and characteristics

**Fig. 1:**
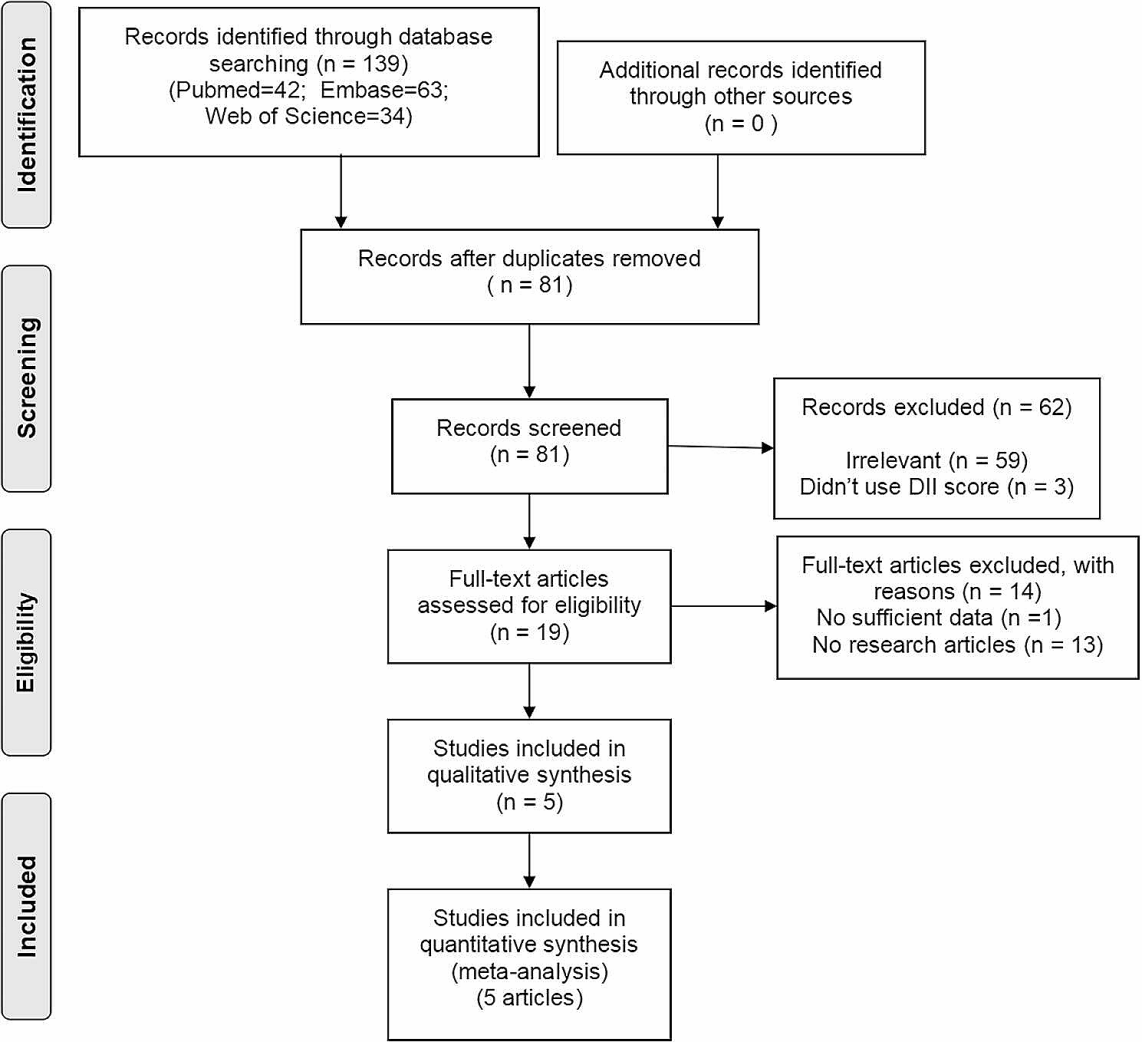
Flow chart of literature search and study selection

A comprehensive literature search was performed as shown in Fig. 1. Initially, 139 papers were identified through database searches. Subsequently, 19 potential studies were retained through reading the titles and abstracts. Afterward, 14 studies were excluded due to insufficient data (1 study) or not being research-oriented articles (13 studies). Finally, 5 articles [27, 28, 38–40] met the inclusion criteria for this meta-analysis.

Table 1 presents the fundamental research information of the included studies. The publication years ranged from 2021 to 2023. In total, these studies contained 197,929 participants with 12,081 COVID-19 cases. Participant sexes included male and female, and ages ranged from 18 to 73 years. All 5 included studies employed the same way of scoring the DII based on a self-reported or face-to-face recorded food frequency questionnaire (FFQ). The rationale and description of the DII methodology were previously published [13]. In brief, the DII was calculated from every inflammatory item in the FFQ. Among them, 4 studies were launched in Iran, while only 1 study was performed in a Western country (the UK). In general, 1 prospective cohort and 4 case-control studies reported the association between DII and the incidence of COVID-19, whereas 2 studies investigated the relation between DII and the severity of COVID-19. There was only 1 study about DII and COVID-19 mortality.

**Table 1:** Baseline characteristics of included studies

Study	Duration	Country	DII Measurement (FFQ)	Median DII score	COVID-19 Cases	Total participants	Mean/ median age (years)	Sex: male (proportion)	OR	95% CI	OR (severity)	95% CI	Adjusted for	Study Quality (NOS score)
Zhao et al. 2023	January 2020 to February 2021	UK	45- items	*−*0.43	11,288	196,154	57	0.553	1.05	1.03–1.06	1.11	1.05–1.16	Age, sex, race/ethnicity, deprivation index, history of heart disease, cancer, type 2 diabetes, smoking status, physical activity, sleep duration	7
Moludi et al. 2021	June to July 2020	Iran	138-items	0.80	60	120	59.3	0.7	3.06	1.07–8.72	1.14	0.95–1.36	Age, sex, BMI, diabetes, blood pressure	6
Firoozi et al. 2022	March 2020 to June 2020	Iran	116-items	-0.51	133	455	40.3	0.547	2.86	2.30–3.55			Age, sex, weight,BMI, waist-to hip circumference rate, educational level, smoking	6
Tavassoli et al. 2023	May 2020 to January 2021	Iran	168-items	-0.08	100	200	39	0.50	1.08	0.92–1.27			Age, sex, diabetes disease, pulmonary disease, BMI	5
Mohajeri et al. 2022	Up to July 2021	Iran	138-items	-1.25	500	1000	41.5	0.51	1.63	1.54–1.72			Age, sex, chronic heart disease, hypertension, diabetes, dysplidaemia, educational level	5

### Quality assessment

Despite potential methodological deficiencies in the quality of the included studies, all were assessed as medium to high quality using the NOS assessment tool, with scores ranging from 5 to 7 (Table 1).

### DII and incidence, severity, and mortality of COVID-19

**Fig. 2:**
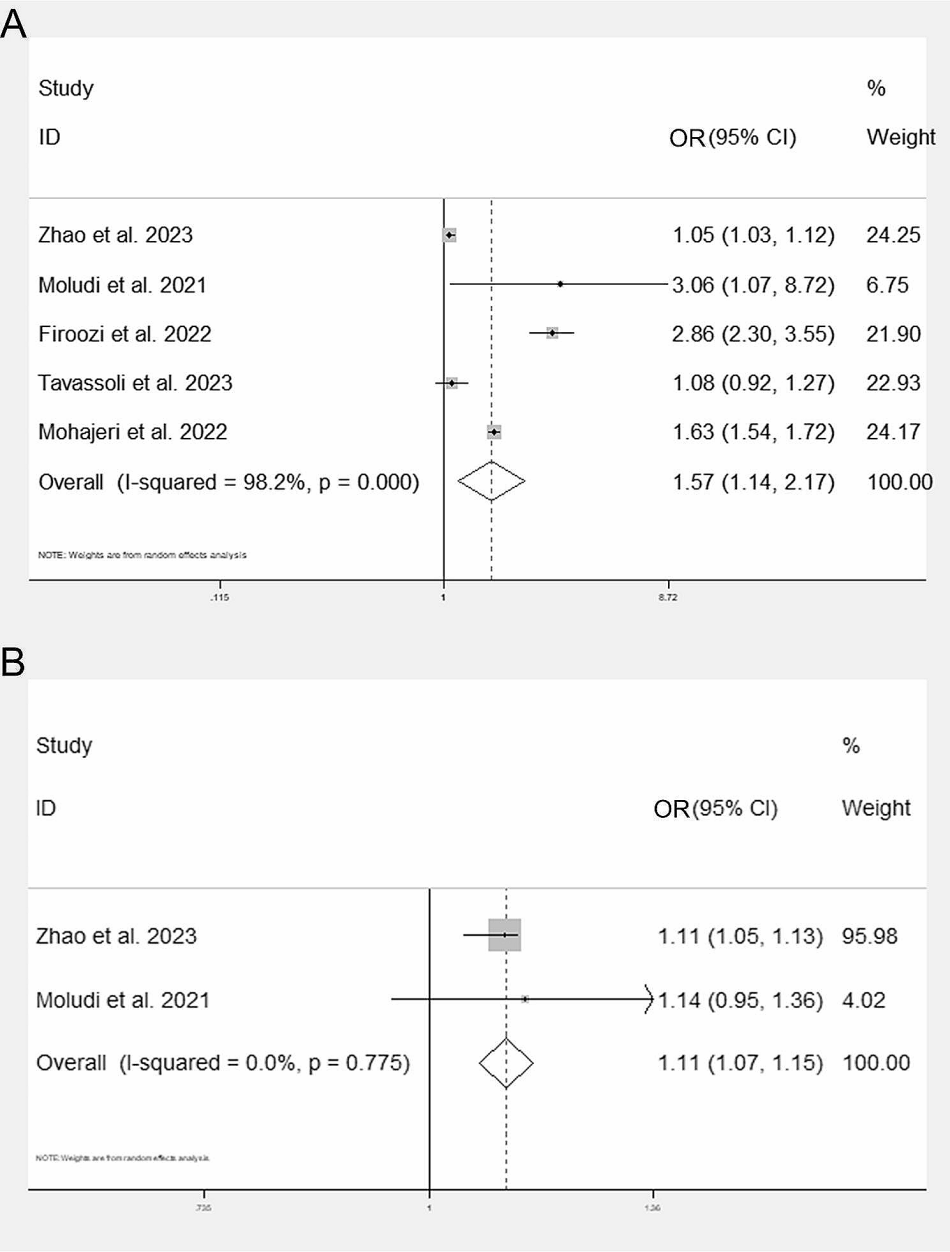
Forest plots of pooled ORs for DII and COVID-19 incidence **(A)** and severity **(B)** OR: odds ratio

As demonstrated in Fig. 2, the forest plot indicates that a higher DII was independently associated with the increasing odds of COVID-19 (pooled OR = 1.57, 95% CI: 1.14, 2.17, Fig. 2A). Only 2 studies investigated the association of DII and COVID-19 severity (defined as the length of hospital-stay in both studies). The result of meta-analysis suggested that elevated DII was also related to more severe COVID-19 (pooled OR = 1.11, 95% CI: 1.07, 1.15, Fig. 2B). Given that only 1 study reported the potential association of DII and the mortality of COVID-19 after our systematic screening, we were unable to do further meta-analysis. Therefore, we adopted the result that the peak value group DII (the highest quintile DII vs. the lowest quintile) was associated with a slightly higher risk ratio (RR) of COVID-19 mortality (RR = 1.43, 95% CI: 1.01, 2.01, *P* = 0.04). However, when considered as a continuous variable, DII was not significantly associated with COVID-19 mortality (RR = 1.13, 95% CI: 1.00, 1.27, *P* = 0.37).

**Fig. 3:**
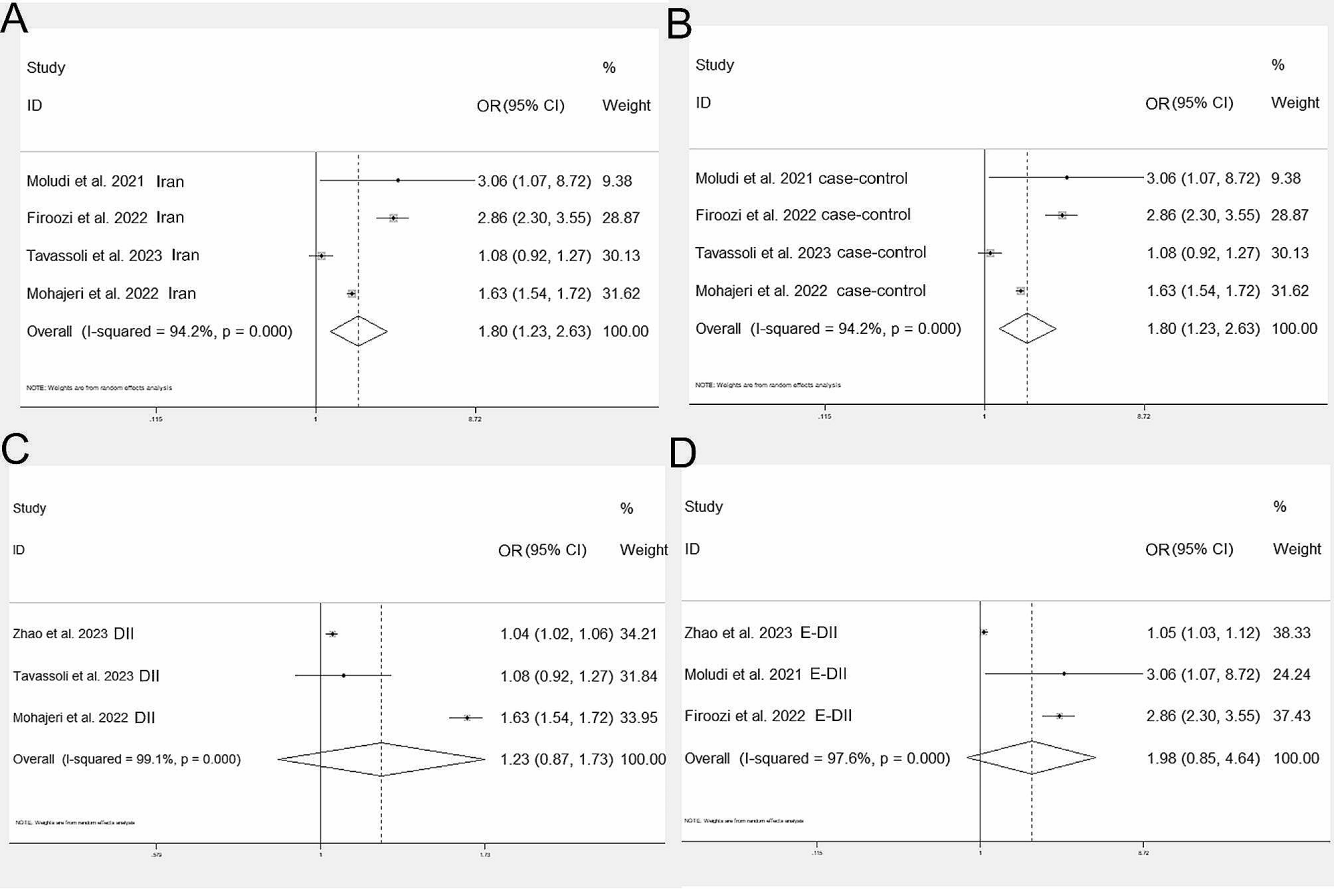
Forest plots of pooled ORs for DII and COVID-19 risk in Asian countries **(A)**, case-control studies **(B)**, DII subgroup **(C)**, energy-adjusted DII group **(D)** OR: odds ratio

Afterwards, we did additional subgroup analyses to explore the association between DII and the infectious risk of SARS-CoV-2 infection incidence. First, we performed subgroup analysis based on different countries. Among the 5 included studies, 4 studies were from an Asian country, Iran. The pooled OR in this group was 1.80 (95% CI: 1.23, 2.63, Fig. 3A). Second, a subgroup analysis was conducted based on study design. There were 4 case-control studies, the pooled OR was 1.80 (95% CI: 1.23, 2.63, Fig. 3B). Third, we respectively performed meta-analysis on DII and energy-adjusted DII. Neither DII (OR = 1.23, 95% CI: 0.87, 1.73, Fig. 3C) nor energy-adjusted DII (OR = 1.98, 95% CI: 0.85, 4.64, Fig. 3D) were significantly associated with the odds of SARS-CoV-2 infection.

### Meta-regression

An obvious heterogeneity was found in the included studies (Fig. 2A, I^2^ = 75.2%), so we performed a meta-regression to seek potential influencing factors. Owing to the insufficient data on other parameters, such as baseline cardiovascular diseases, we chose sample size and sex proportion as covariates to estimate between-study variance. The results of this regression model indicated that the 2 factors above (sample size: *P* = 0.292, sex proportion: *P* = 0.310) were not the direct source of heterogeneity.

### Dose-response analysis

**Fig. 4:**
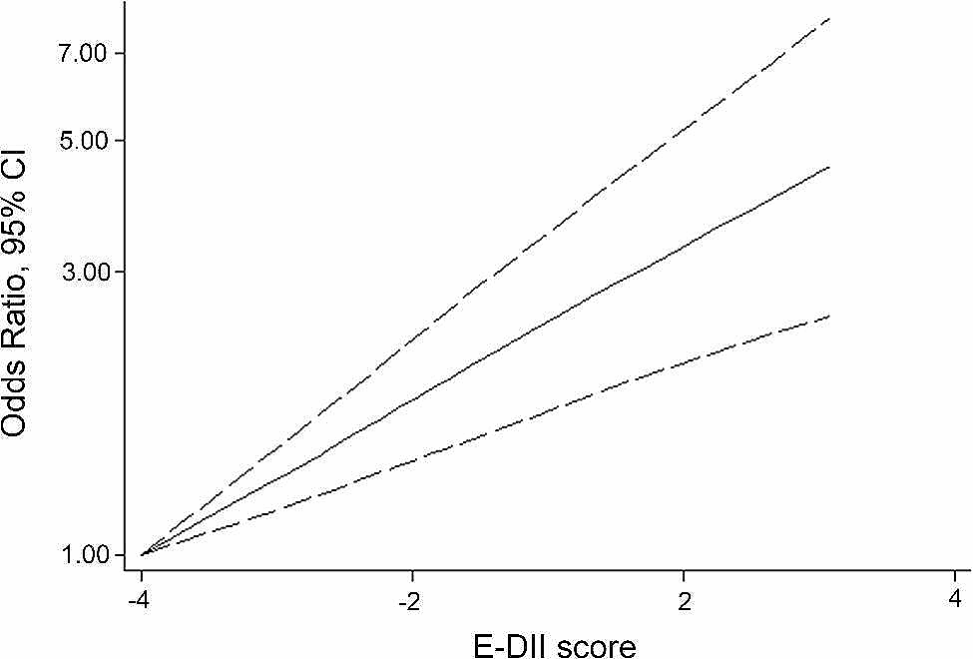
Dose-response analysis plots of energy adjusted DII (E-DII) and COVID-19 incidence. Full line: estimated odds ratio; dashed line: 95% confidence interval of odds ratio

As shown in the dose-response plot, the pooled dose-response OR of the non-linear model was 1.31 (95% CI: 1.20, 1.43, Z value = 5.91, *P* < 0.001, Fig. 4), demonstrating that human COVID-19 risk increased by 31% after E-DII increasing by 1-point increase in the score. Unfortunately, dose-response analysis was not conducted on the severity or mortality of COVID-19 due to the lack of original data.

### Sensitivity analysis

**Fig. 5:**
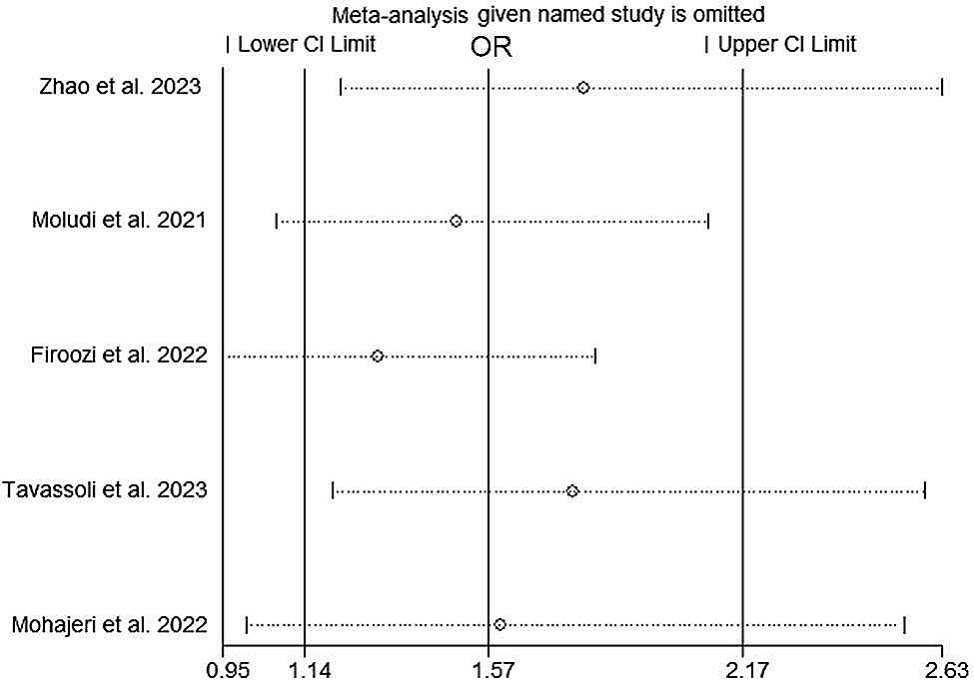
Sensitivity analysis of included studies

A sensitivity analysis was performed by removing 1 study in sequence to test the stability of our pool results. The removal of a single study did not significantly impact the main results, as shown in Fig. 5.

### Publication bias

**Fig. 6:**
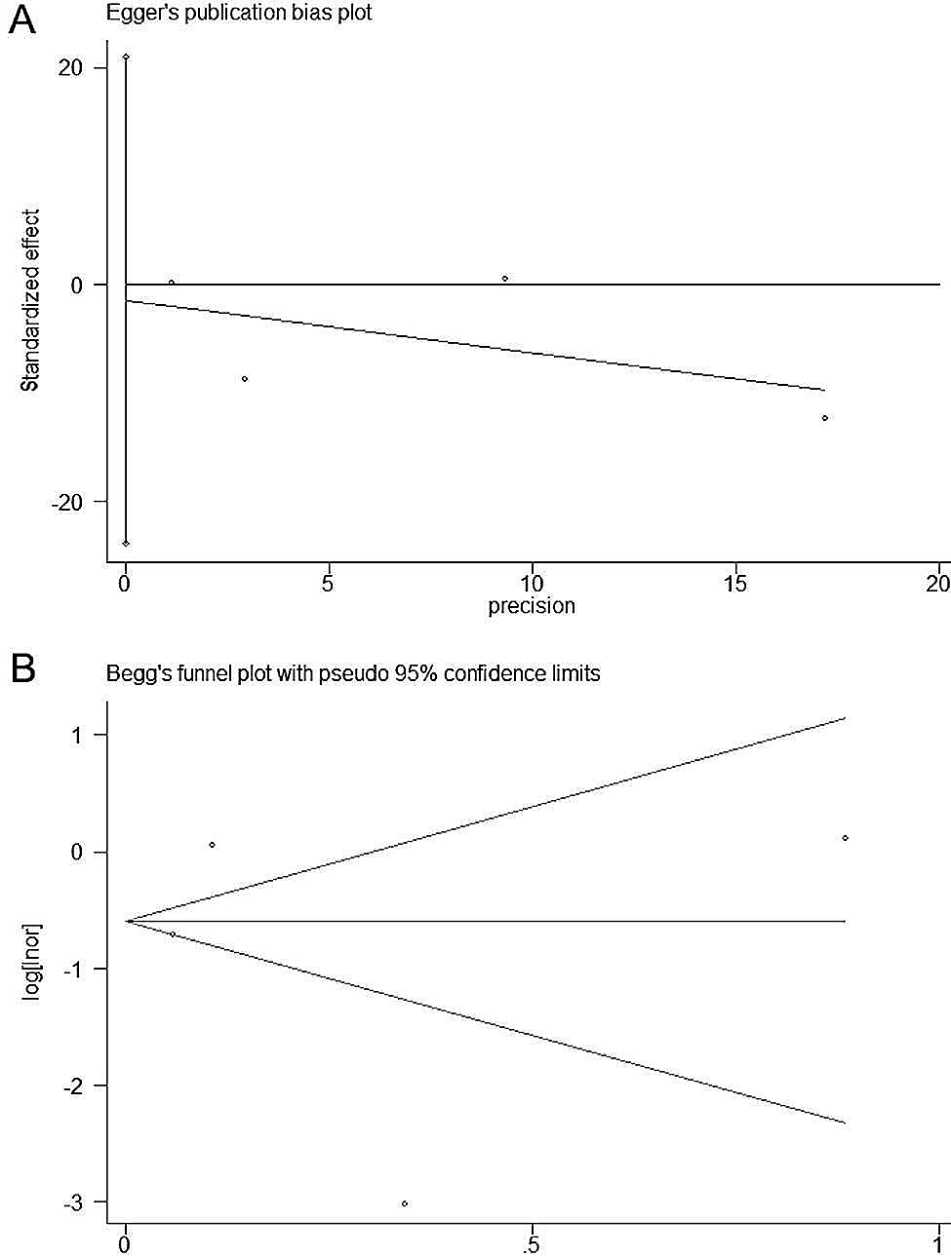
Plots of Egger’s test **(A)** and Begg’s test **(B)**

In order to gauge publication bias, we performed both Egger’s and Begg’s tests. Neither the Egger’s test (*P* = 0.301, > 0.1) nor the Begg’s test (*P* = 0.933, > 0.1) indicated potential publication bias in the association between DII and COVID-19 risk. The corresponding plots of the upon tests are displayed in Fig. 6.

## Discussion

In this study, we systematically summarized the existing evidence from the 5 included studies with 197,929 participants. The results indicate that a high DII score is independently associated with an increased incidence of SARS-CoV-2 infection incidence (OR = 1.57, 95% CI: 1.14, 2.17). Additionally, individuals with an elevated DII score had an 11% increased odds ratio of COVID-19 hospital-stay severity compared to those with a lower DII. Although individuals with the highest quintile DII may have a tiny increased risk of COVID-19 deaths, we should not draw the conclusion that DII is associated with the mortality of COVID-19 based on current evidence. Importantly, we objectively concluded that the incidence of SARS-CoV-2 infection increased by 31% for each 1-point increase in the E-DII based on a non-linear dose-response analysis. However, the correlation of high DII and COVID-19 should be interpreted with caution. Subgroup analyses based on study design and different countries did not significantly affect the main results. Although the energy-adjusted DII subgroup showed a similar rising trend with higher COVID-19 incidence, the results in this group were not statistically significant. We consider that the obvious heterogeneity among studies may account for this situation to some extent. Different SARS-CoV-2 variants and various local lockdown prevention policies may contribute to the heterogeneity. Based on all the aforementioned results, we believe that DII can be utilized as an objective and reliable tool in evaluating dietary inflammatory potential and preventing COVID-19.

A pro-inflammatory condition is positively correlated with COVID-19, potentially leading to increased nitrogen species and reactive oxygen levels, serving as a crucial factor in the development of COVID-19 severity [5]. Viral infection and replication can lead to inflammation through cytopathic effect directly [41]. During this period, there is an upregulation of pro-inflammatory cytokines and chemokines, leading to the recruitment of monocytes, eosinophils, and T lymphocytes. This phenomenon can explain the state of lymphocytopenia observed in some patients with COVID-19 [42]. Furthermore, in the cases of dysregulated immune response, elevated levels of inflammatory cytokine secretion such as interferon-gamma (IFN-γ), monocyte chemo-attractant protein-1 (MCP-1) and interleukin-6 (IL-6) may lead to an excessive inflammatory response, known as cytokine storm [43, 44].

Interestingly, researchers observed a reduction in the consumption of high DII foods, such as fast food, soft drinks, and alcoholic beverages during the COVID-19 pandemic, which was probably reduced by mobility restrictions and social gatherings [45]. In view of the above, this study may be timely and worthwhile. It suggests that promoting a healthy anti-inflammatory dietary pattern with a lower Dietary Inflammatory Index (DII) score could be a non-pharmacological approach to prevent COVID-19.

Despite conducting the first dose-response meta-analysis on DII and COVID-19, there are still several limitations. First, the publication language was not restricted, but all the 5 studies included were written in English. We identified no relevant articles published in Chinese or other languages which were qualified for the inclusion criteria. An extent of language bias may exist. Second, although supported the main results and there was no evidence of publication bias, the number of included studies was small in some subgroups. For instance, only 2 studies provided data on and DII and the severity of COVID-19. Moreover, the methodology had some limitations. Therefore, we emphasize the need for careful interpretation of the results. Third, within the 5 included studies, 4 were case-control studies, in which recall bias might exist. As a matter of fact, participants from only 1 prospective cohort also fulfilled the FFQ in an electronic self-reported way rather than a face-to-face solid-recorded interview, which may lead to unstable or exaggerated results. Fourth, the 5 included studies were from Iran (4 studies) or the UK (1 study). No studies on DII and COVID-19 from America, Oceania, East-Asian, or Africa were found to date. Therefore, further large prospective cohort studies in diverse populations are urgently needed.

In conclusion, this dose-response meta-analysis reveals that elevated DII is associated with a higher infectious incidence and severity of COVID-19, with the SARS-CoV-2 infectious odds increasing by 31% for each 1-point increase in the Enhanced DII (E-DII) score. However, there is insufficient evidence on COVID-19 mortality. Therefore, large prospective cohort studies in diverse regions or countries are warranted to validate the findings of this study.

### Electronic supplementary material

Below is the link to the electronic supplementary material.

Supplementary Material 1

## Data Availability

This is a meta-analysis, and all data in this article were in published articles.

## Declarations

### PROSPERO Registry number and websites

PROSPERO :**CRD42023407410** Available from: https://www.crd.york.ac.uk/prospero/display_record.php?RecordID=407410
